# Management of intracranial tuberculous mass lesions: how long should we treat for?

**DOI:** 10.12688/wellcomeopenres.15501.3

**Published:** 2020-02-26

**Authors:** Suzaan Marais, Ronald Van Toorn, Felicia C. Chow, Abi Manesh, Omar K. Siddiqi, Anthony Figaji, Johan F. Schoeman, Graeme Meintjes, Tuberculous Meningitis International Research Consortium, Rob E. Aarnoutse, Rob E. Aarnoutse, Suzanne T. B. Anderson, Suzanne T. B. Anderson, Nathan C. Bahr, Nguyen D. Bang, David R. Boulware, Tom Boyles, Lindsey H. M. te Brake, Satish Chandra, Felicia C. Chow, Fiona V. Cresswell, Reinout van Crevel, Angharad G. Davis, Sofiati Dian, Joseph Donovan, Kelly E. Dooley, Anthony Figaji, A. Rizal Ganiem, Ravindra Kumar Garg, Diana M. Gibb, Raph L. Hamers, Nguyen T. T. Hiep, Darma Imran, Akhmad Imron, Sanjay K. Jain, Sunil K. Jain, Byramee Jeejeebhoy, Jayantee Kalita, Rashmi Kumar, Vinod Kumar, Arjan van Laarhoven, Rachel P-J. Lai, Abi Manesh, Suzaan Marais, Vidya Mave, Graeme Meintjes, David B. Meya, Usha K. Misra, Manish Modi, Alvaro A. Ordonez, Nguyen H. Phu, Sunil Pradhan, Kameshwar Prasad, Alize M. Proust, Lalita Ramakrishnan, Ursula Rohlwink, Rovina Ruslami, Johannes F. Schoeman, James A. Seddon, Kusum Sharma, Omar Siddiqi, Regan S. Solomons, Nguyen T. T. Thuong, Guy E. Thwaites, Ronald van Toorn, Elizabeth W. Tucker, Sean A. Wasserman, Robert J. Wilkinson

**Affiliations:** 1 Department of Neurology, Inkosi Albert Luthuli Central Hospital and University of KwaZulu-Natal, Durban, 4091, South Africa; 2 Division of Neurology, Department of Medicine, Groote Schuur Hospital and University of Cape Town, Cape Town, 7925, South Africa; 3 Department of Pediatrics and Child Health, Stellenbosch University, Cape Town, 7505, South Africa; 4 Weill Institute of Neurosciences and Department of Neurology and Division of Infectious Diseases, University of California, San Francisco, California, 94110, USA; 5 Department of Infectious Diseases, Christian Medical College, Vellore, 632004, India; 6 Department of Neurology, Beth Israel Deaconess Medical Center, Boston, Massachusetts, 02215, USA; 7 Department of Internal Medicine, University of Zambia School of Medicine, Lusaka, Zambia; 8 Division of Neurosurgery and Neuroscience institute, University of Cape Town, Cape Town, 7700, South Africa; 9 Wellcome Centre for Infectious Diseases Research in Africa, Institute of Infectious Disease and Molecular Medicine, Department of Medicine, University of Cape Town, Cape Town, 7925, South Africa

**Keywords:** tuberculosis, central nervous system, treatment duration, management, imaging, tuberculous meningitis, tuberculoma, tuberculous abscess

## Abstract

Tuberculous intracranial mass lesions are common in settings with high tuberculosis (TB) incidence and HIV prevalence. The diagnosis of such lesions, which include tuberculoma and tuberculous abscesses, is often presumptive and based on radiological features, supportive evidence of TB elsewhere and response to TB treatment. However, the treatment response is unpredictable, with lesions frequently enlarging paradoxically or persisting for many years despite appropriate TB treatment and corticosteroid therapy. Most international guidelines recommend a 9-12 month course of TB treatment for central nervous system TB when the infecting *Mycobacterium tuberculosis* ( *M.tb*) strain is sensitive to first-line drugs. However, there is variation in opinion and practice with respect to the duration of TB treatment in patients with tuberculomas or tuberculous abscesses. A major reason for this is the lack of prospective clinical trial evidence. Some experts suggest continuing treatment until radiological resolution of enhancing lesions has been achieved, but this may unnecessarily expose patients to prolonged periods of potentially toxic drugs. It is currently unknown whether persistent radiological enhancement of intracranial tuberculomas after 9-12 months of treatment represents active disease, inflammatory response in a sterilized lesion or merely revascularization. The consequences of stopping TB treatment prior to resolution of lesional enhancement have rarely been explored. These important issues were discussed at the 3 ^rd^ International Tuberculous Meningitis Consortium meeting. Most clinicians were of the opinion that continued enhancement does not necessarily represent treatment failure and that prolonged TB therapy was not warranted in patients presumably infected with *M.tb* strains susceptible to first-line drugs. In this manuscript we highlight current medical treatment practices, benefits and disadvantages of different TB treatment durations and the need for evidence-based guidelines regarding the treatment duration of patients with intracranial tuberculous mass lesions.

## Disclaimer

The views expressed in this article are those of the author(s). Publication in Wellcome Open Research does not imply endorsement by Wellcome.

## Introduction

Neurological tuberculosis (TB) manifests as meningitis, radiculomyelitis, bony spinal disease and tuberculoma/tuberculous abscess that may occur intracranially or within the spinal space ^1^. Similar to the other neurological TB manifestations, tuberculous mass lesions are common in settings with high TB incidence ^2, 3^, and high HIV prevalence ^4– 7^, where this diagnosis accounts for a significant proportion of intracranial space occupying lesions. The diagnosis of intracranial tuberculoma is most often presumptive and based on radiological features, supportive evidence of TB elsewhere and response to TB treatment. However, the treatment response of tuberculomas is unpredictable and lesions may persist for many years despite appropriate TB treatment and adjunctive corticosteroid therapy ^8– 12^. The optimal duration of TB treatment is unknown and clinical practice varies. In this manuscript we highlight current divergent clinical practice, benefits and disadvantages of different TB treatment durations and the need for prospective clinical trial data to determine the optimal treatment duration in patients with intracranial tuberculous mass lesions.

### Pathogenesis and pathology

Hematogenous seeding after the primary infection is one proposed mechanism of central nervous system (CNS) involvement in TB ^13^. Miliary disease may increase the risk of hematogenous spread to the CNS ^14^. *Mycobacterium tuberculosis* ( *M.tb*) may enter the CNS via direct infection of endothelial cells or trafficking through infected phagocytes ^15, 16^, which is followed by the formation of tubercles, most commonly in the brain cortex or meninges. Rupture of an adjacent tubercle into the subarachnoid space results in tuberculous meningitis (TBM), whilst tubercles that do not rupture may progress to form tuberculomas ^13^. Tuberculomas show granulomatous inflammation with a central area of caseous necrosis surrounded by epithelioid histiocytes, Langerhan’s giant cells, lymphocytes, astrocytes and vascular proliferation that evolves to develop a thick vascular connective tissue layer.

The mycobacterial burden in CNS TB is low. The impressive pathology and evolution of lesions during TB therapy highlights the role of the host inflammatory response in pathogenesis. Microglia in the CNS are infected by *M.tb* and activated microglia release many cytokines that play a crucial role in pathogenesis ^17^. TNF-α is a central molecule in the control and mediation of inflammation in CNS TB. While TNF-α is involved in granuloma formation and control of disease, elevated levels are associated with markers of increased pathology such as cerebrospinal fluid leukocytosis, higher levels of other soluble inflammatory mediators, increased *M.tb* load and clinical deterioration ^18^. Studies focused on the vasculature associated with tuberculomas have revealed significant vasculitis with proliferative changes in the basement membrane ^19^.

Occasionally, tubercles may coalesce or continue to progress to form a tuberculous abscess, which is a large pus-filled encapsulated lesion containing bacilli ^20, 21^. Histopathologically, the tuberculous abscess wall shows chronic vascular granulation tissue whilst lacking the granulomatous reaction of a tuberculoma.

### Clinical presentation

The clinical features of tuberculomas depend on their anatomic location in the brain, related to local mass effect, obstruction of cerebrospinal fluid pathways, and/or seizures. Supratentorial lesions are common in adults while infratentorial involvement is slightly more common in children ^22^. Patients usually present sub-acutely with symptoms and signs such as headaches, seizures, depressed level of consciousness, and focal neurological deficits ^12, 23, 24^. Infratentorial lesions commonly present with hydrocephalus. Pituitary apoplexy and movement disorders like chorea are rare manifestations of tuberculomas ^25, 26^. If associated with TBM, meningeal symptoms and signs may dominate the clinical picture. Tuberculous abscesses have a more accelerated course, often presenting acutely with associated fever ^21^.

### Imaging findings

**Figure 1.**
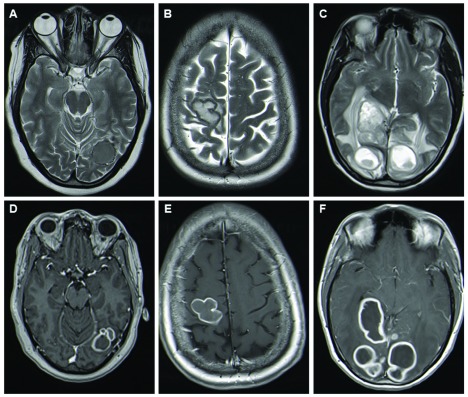
Magnetic resonance imaging of various categories of tuberculous mass lesions. Axial T2-weighted images ( **A**, **B** and **C**) and corresponding T1-weighted post-contrast images ( **D**, **E** and **F**) of caseating solid tuberculoma (A and D), caseating liquid tuberculoma ( **B** and **E**) and tuberculous abscess ( **C** and **F**).

Neuroimaging is essential for identifying intracranial tuberculous mass lesions with findings determined by the composition of the lesion. Tuberculomas have classically been categorized as non-caseating, caseating solid, and caseating liquid, that can be differentiated on computed tomography (CT) and magnetic resonance imaging (MRI) ( Figure 1) ^21^. Multiple lesions are seen more often than isolated lesions though the latter is still common ^27, 28^. Perilesional edema can be present or absent.

CT is the most frequent modality used to identify tuberculomas due to its wide availability though it has limitations in resolution. Tuberculomas typically appear as round or lobulated nodules that are hypodense or isodense to the brain parenchyma. CT with contrast most commonly shows rim enhancement of lesions but nodular or homogeneous enhancement can also be seen ^12^.

MRI is the preferred modality for the identification of tuberculomas due to superior resolution and better visualization of the posterior fossa relative to CT. Non-caseating granulomas are hypointense or isointense on T1-weighted imaging (T1WI) and hyperintense on T2-weighted imaging (T2WI, “T2-bright”) with homogeneous contrast enhancement ^21^. Caseating solid granulomas are hypointense or isointense on T1WI and hypointense on T2WI (“T2-black”) with rim enhancement. Caseating liquid granulomas, which are rare, are hypointense on T1WI and hyperintense on T2WI with rim enhancement. Tuberculous abscesses may be indistinguishable from tuberculomas with a liquid center on standard MRI settings, but they are usually larger (>3 cm in diameter) and thin-walled in appearance ( Figure 1) ^21^. Miliary tuberculomas appear as multiple, small (2–3 mm), scattered lesions that typically rim enhance with contrast administration and lack perilesional edema ^29^.

**Figure 2.**
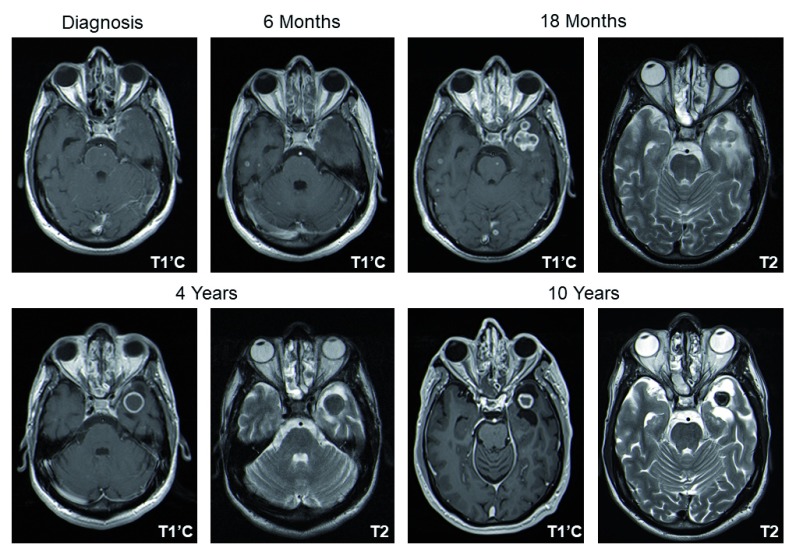
Serial magnetic resonance imaging of a patient with drug-susceptible central nervous system tuberculosis who received TB treatment for 4 years. Axial T1-weighted post-contrast (T1’C) images and T2-weighted (T2) images are shown. At diagnosis, a miliary pattern with focal meningeal enhancement of the left temporal lobe was noted, which persisted at 6-months follow-up. At 18 months, a lobulated rim-enhancing tuberculoma had developed in the left temporal lobe which was of mixed intensity on T2-weighted images with surrounding edema. Despite gradual reduction in lesion size and perilesional edema with associated atrophy, rim-enhancement persisted during the next 8.5 years of follow-up. Notably, the patient did not deteriorate clinically after cessation of TB treatment and the T2-signal of the lesion became increasingly hypointense (“T2-Black”) suggesting cure.

**Figure 3.**
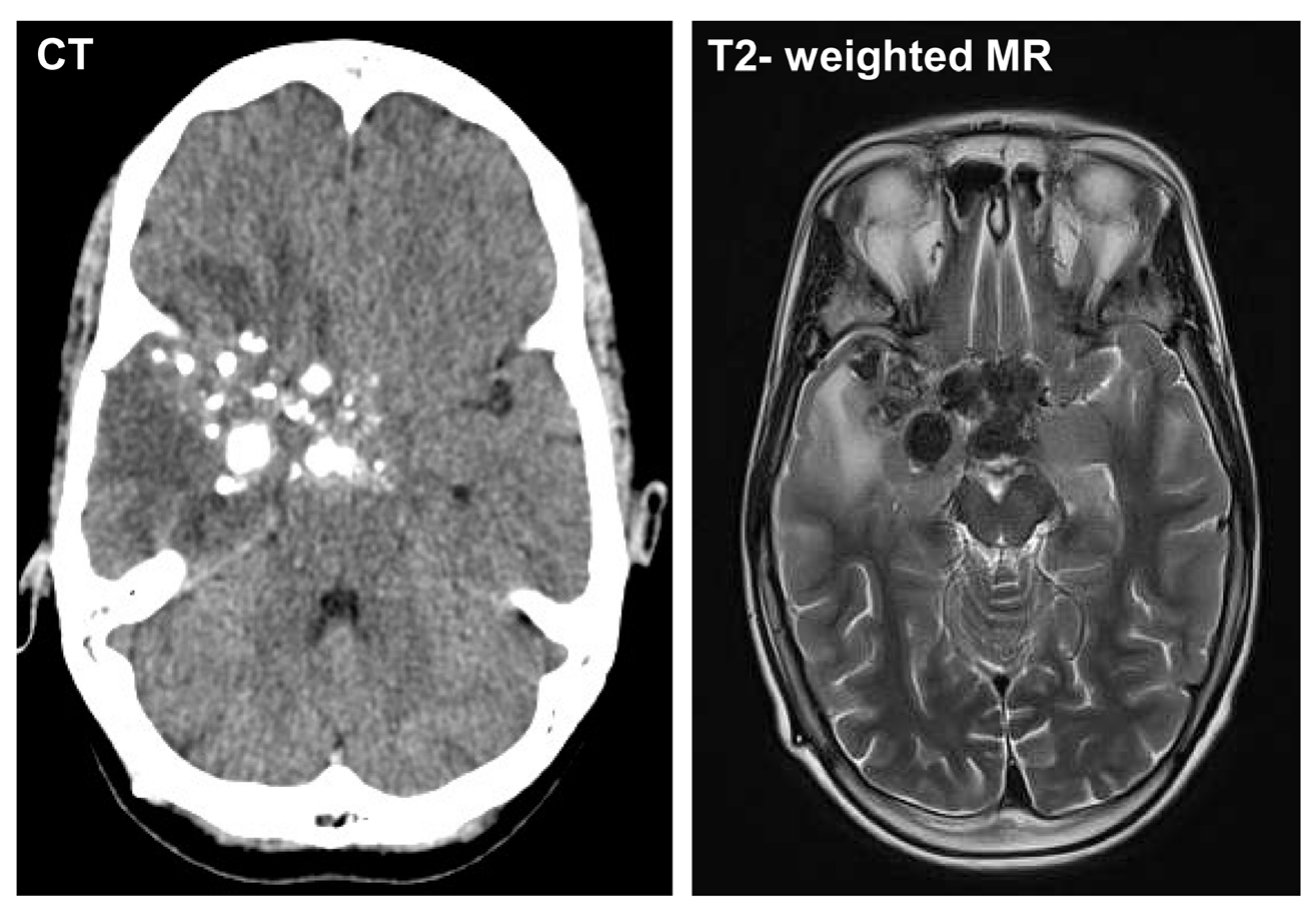
Corresponding magnetic resonance (MR) and computed tomography (CT) images of tuberculomas demonstrating calcification by CT imaging of lesions that appear hypointense (“T2-black”) by T2-weighted MRI.

Evidence of a satisfactory radiological response on serial brain imaging after TB treatment initiation includes a reduction in perilesional edema, decrease in lesion size and calcification (seen on CT). Other findings supportive of improvement of liquified tuberculomas and abscesses on MRI are a decrease in T2 brightness and, subsequently, loss of T2 signal. Evolution of TB abscesses from early-stage “T2-bright” with edema to “T2-black” lesions may represent a marker for cure ( Figure 2) ^30^. In our experience, the resultant homogeneous “T2-black” tuberculoma (with rim T1 contrast enhancement) may persist for many months in asymptomatic patients without relapse off TB treatment. CT of such lesions usually shows gradual calcification, which most often involves the capsule ( Figure 3).

The differential diagnosis of enhancing brain lesions is vast and includes infective, neoplastic, inflammatory, demyelination and vascular conditions that may be indistinguishable from tuberculous mass lesions by CT and conventional MRI sequences ^31, 32^. Although the presence of a "target sign" on CT, which consists of a rim enhancing lesion with central calcification, is highly suggestive of a tuberculoma ^33, 34^, it is uncommon and other conditions may mimic these findings ^35^. MRI techniques such as diffusion weighted imaging (DWI) may improve the diagnostic accuracy of MRI for evaluating patients with intracerebral tuberculous mass lesions ^36^. DWI is a technique that characterizes tissues on the basis of the molecular motion of water contained within them. Tuberculous lesions that show hyperintense centers on T2WI (i.e. caseating liquid granulomas and tuberculous abscesses) frequently show increased signal intensity cores on DWI and have reduced apparent diffusion coefficient (ADC) values, which are consistent with restricted diffusion within the lesion ^37, 38^. Conversely, caseating solid tuberculomas (hypointense centers on T2WI) most commonly show decreased signal intensity cores on DWI ^37^ and have significantly higher ADC values compared to T2 hyperintense lesions ^38, 39^. As caseating liquid tuberculomas are rare, most tuberculomas do not demonstrate restricted diffusion. One of the main applications of DWI in tuberculous mass lesions is its ability to distinguish those with restricted diffusion from necrotic and cystic tumors, that typically show features consistent with free diffusion ^40, 41^. However, tuberculous brain abscesses are indistinguishable from pyogenic abscesses that also typically show reduced diffusion ^36, 41^. It may be possible to distinguish tuberculous lesions with restricted diffusion from other infections such as neurocysticercosis ^38, 39^, cryptococcomas ^36^ and toxoplasmosis ^36^, that do not typically show restricted diffusion. However, DWI findings is variable in the vast majority of infective causes of intracerebral lesions ^36^ and results should therefore be considered in conjunction with clinical and other investigation findings. Other advanced MRI techniques such as MR spectroscopy (MRS), magnetized transfer (MT) imaging and susceptibility-weighted imaging (SWI) are also under investigation to improve the diagnostic yield of tuberculous brain lesions, but are beyond the scope of this article ^34, 39^.

### Paradoxical reactions

Paradoxical enlargement or the development of new intracranial tuberculomas or abscesses in patients with CNS or extra-neural TB on appropriate treatment is well-described ^8, 42– 54^. Such reactions typically occur within the first six months after TB treatment initiation ^45, 51, 52, 55^, but may rarely be delayed for a year or more ^10, 55– 57^. Paradoxical reactions are often identified when patients present with neurological deterioration during TB treatment, prompting brain imaging. In case series of predominantly HIV-uninfected patients with CNS TB, clinical deterioration due to paradoxical tuberculoma reaction has been described in 6–29% ^8, 42– 49^. However, many of these patients are asymptomatic during these episodes and the frequency of detecting paradoxical tuberculoma development or enlargement increases substantially (from 29% to 65%) if surveillance brain imaging is performed during the first six months of TB treatment ^45^. Paradoxical TB reactions are more common in HIV-infected patients, particularly in those who commence antiretroviral therapy (ART) after starting TB treatment, in which case it is referred to as paradoxical TB-immune reconstitution inflammatory syndrome (TB-IRIS) ^58– 61^. The influence of HIV on the frequency of paradoxical tuberculoma reactions (separate from the effect of ART) has rarely been reported. One recent study of 47 HIV-infected and 14 HIV-uninfected adults with tuberculomas found no difference in the frequency of paradoxical reactions by HIV status (36% in each group) ^12^. The majority of HIV-infected patients were receiving ART prior to tuberculoma presentation or did not start ART after diagnosis, precluding the development of TB-IRIS in this group. The pathogenesis of paradoxical reactions (including IRIS) remains unclear but is likely related to an aberrant immune response to TB antigens rather than failure of TB treatment ^52, 62^. Clinical findings supporting this view are the observation that new or enlarging tuberculomas in TBM patients frequently appear in those known to be infected with drug-susceptible strains who show clinical and radiological improvement of other aspects of TBM ( Figure 2) ^53^. Another argument is that anti-inflammatory drugs (corticosteroids and thalidomide) are effective in the prevention and management of paradoxical TB reactions, including tuberculomas ^53, 63– 65^. Anecdotal case reports describe patients who develop symptomatic intracranial tuberculoma months to years after completion of 12 months to four years of TB treatment for neurological TB ^9, 30, 66, 67^. Whether some of these cases represent disease reactivation, an inadequate TB treatment duration or infection with a drug-resistant strain is uncertain and patients are often empirically recommenced on TB treatment, either with first-line drugs or a multidrug-resistant regimen, at time of deterioration ^9, 30, 66^. However, a delayed paradoxical response responsive to corticosteroids without additional TB treatment accounts for at least some of these cases ^67^.

### Medical treatment

The mainstay of treatment of intracranial tuberculomas is similar to that of TBM and includes TB therapy and corticosteroids. The World Health Organization, Centers for Disease Control and Prevention of America and the British Thoracic Society recommend a 9–12 month course of TB treatment for CNS TB when the *M.tb* strain is sensitive to all drugs ^68– 70^. However, these guidelines are based on expert opinion rather than randomized controlled trials. Specifically, no studies have compared different treatment durations in patients with intracranial tuberculomas. The morphology of the lesion plays an important role in response to therapy and a one-size-fits-all approach may therefore be inappropriate in the decision regarding tuberculoma treatment duration. This is suggested by the almost invariably good response of miliary tuberculomas to TB treatment (presumably non-caseous) and the frequent persistence of caseous and liquified TB lesions (e.g. abscesses) despite TB treatment ^53, 71^.

Some guidelines suggest adjunctive systemic corticosteroids in all forms of CNS TB, including those in whom a strong suspicion of tuberculoma exists ^70^. Corticosteroid therapy may be of particular value when there is significant perilesional edema (resulting in symptomatology) and in cases where there is paradoxical enlargement despite optimal TB therapy ^72^. Corticosteroid duration should be tailored according to the radiological response of the tuberculoma and clinical wellbeing of the patient and balanced against side effects.

TB abscesses are often unresponsive to standard TB therapy with corticosteroids. Although no clinical trials exist, adjuvant thalidomide therapy (3–5 mg/kg/day) has been shown to be beneficial in patients who develop enlarging TB abscesses ^53^. In our experience, thalidomide can be stopped without relapse when clinical improvement is optimal or reached a plateau, regardless of whether radiological resolution has been achieved. Other immunomodulatory agents that have anecdotally been beneficial in steroid resistant paradoxical TB reactions affecting the CNS include the TNF-α inhibitor infliximab ^73^ and interferon-γ ^74^. The therapeutic benefit of these and other host-directed therapies aimed at reducing excessive CNS inflammation should be investigated in future prospective studies.

### Surgical management

There are no controlled studies to determine the role of surgery in patients with intracranial tuberculous mass lesions. However, there are general principles from clinical practice and the existing literature that can be summarized ^10, 75^. Biopsy for diagnosis is considered: 1) at the outset if the definitive diagnosis is unclear, and 2) for persistence or paradoxical growth of a presumed tuberculoma despite medical treatment (for diagnostics and drug sensitivity testing). Resection of the lesion may be considered: 1) to relieve symptomatic or potentially life-threatening mass effect and/or hydrocephalus, and 2) to treat medically refractory seizures. Drainage of abscesses is considered for symptomatic mass effect or hydrocephalus, especially when large and/or in the posterior fossa. However, surgery for tuberculous mass lesions is rarely performed in TB endemic settings as the clinical and imaging information is usually sufficient to make the diagnosis. Furthermore, risks associated with surgery, especially if the lesion is located in an eloquent or difficult to access brain area, and inadequate neurosurgical facilities usually combine to preclude surgical management.

### Duration of TB treatment: what happens in practice?

There is variation in opinion and practice with respect to the duration of TB treatment in patients with intracranial tuberculomas or tuberculous abscesses. A major reason for this is the lack of prospective clinical trial evidence. In rare cases where a microbiological diagnosis is achieved, it is not feasible to access repeated clinical specimens from the site of disease to ascertain whether and when culture conversion has occurred, unlike pulmonary TB where sputum *M.tb* culture can be monitored and treatment duration adjusted accordingly. Monitoring is performed clinically and with brain imaging, with the frequency of both clinical and radiological follow-up being highly variable, depending on factors such as available resources, clinician’s preference and individual patient characteristics ^12^.

The routine duration of TB treatment in intracranial tuberculoma cases include periods of 6 ^76^, 9 ^24, 42, 50, 77^, 12 ^78^, 15 ^49^ and 18 ^9, 10, 23, 45, 79, 80^ months depending on the clinician’s preference. Table 1 presents duration of treatment and outcome in tuberculoma studies published in English ^8, 9, 11, 12, 23, 24, 42, 43, 46, 56, 71, 78, 79, 81– 86^. Although some studies describe radiological resolution of tuberculoma in more than 80% of patients after 6–12 months of TB treatment ^43, 76, 78, 83, 84^, others have reported persistently enhancing lesions in the vast majority (71–82%) of cases after 9–12 months of treatment ^23, 42^. Even after 24 months of therapy, tuberculomas may persist in 22%–46% of cases ^9, 12, 23^ ( Figure 2). Larger lesions (>2.5 cm) are significantly more likely to persist after 18 to 24 months of treatment ^9, 12^. The medical management of patients with persistent intracranial tuberculoma after a “complete treatment course” (6–18 months) is particularly controversial. Some experts suggest continuing treatment until radiological resolution of enhancing lesions has been achieved ^23, 87^, which may unnecessarily expose patients to potentially toxic drugs for many years ^8, 9, 11, 12, 23, 78, 81, 88^; in a study from South Africa, more than 50% of tuberculoma patients followed for 9 months or more (31/57) received TB treatment for more than 18 months (range 19–46 months) ^12^. Others are of the opinion that lesional persistence beyond 18 months does not reflect treatment failure, but rather represents a persistent immune response at the disease site that has been sterilized, hence extending TB treatment beyond this period will not add any benefit ^89^.

**Table 1.** Summary of reported medical management strategies and clinical and radiologic outcomes of intracranial tuberculoma case series.

Study, First author, year published, country	Study design	Patients, n (age group) ^1^	Duration of ATT, Months: %	Steroid use,%	Favorable clinical outcome, %, (n/N) ^2^	Radiologic persistent tuberculoma(s), % (n/N): months F/U
Afghani ^81^, 1994, multiple	Case report + review	41 (C + A)	10-24:100	80 ^3^	68 (25/37)	N/A
Anuradha ^42^, 2011, India	Retrospective observational	43 (C + A)	9: 100	100	26 (11/43)	79 (30/38): 9
Awada ^43^, 1998, Saudi Arabia	Retrospective observational	18 (C + A)	12-18: 100	67	N/A	100 (18/18): 12
Bayindir ^82^, 2006, Turkey	Retrospective observational	23 (C + A)	12-18: 100	N/A	100 (15/15)	N/A
Gupta ^83^, 1990, India	Prospective observational	31 (C + A)	11-12: 97	N/A	N/A	14 (4/29): 12
Gupta ^8^, 2003, India	Prospective observational	9 (C + A)	16: 11 18-34: 88	89	44 (4/9)	N/A
Harder ^84^, 1983, Saudi Arabia	Retrospective observational	20 (C + A)	12: 61 9-24: 39 ^4^	75	35 (7/20)	0 (0/10): 12 ^5^
Idris ^79^, 2007, Sudan	Retrospective observational	16 (A)	18: 100 ^6^	56	N/A	13 (2/16): 18
Li ^80^, 2012, China	Retrospective observational	6 (A)	18: 100	33	83 (5/6)	N/A
Man ^46^, 2010, France	Retrospective observational	23 (A)	9-18: 88 21: 12 ^4^	43	53 (10/19)	75 (12/16): 9-21
Marais ^12^, 2019, South Africa	Retrospective observational	66 (A)	≥9: 96% 19-46: 54 ^4^	76	37 (20/54)	49 (20/41): 18 33 (14/42): 24
Nair ^9^, 2019, India	Retrospective observational	86 (C + A)	≥18: 100 >24-120: 22	N/A	N/A	22 (19/86): 24
Poonnoose ^23^, 2003, India	Retrospective observational	28 (C + A)	≥18: 100	54	68 (19/28)	69 (19/28): 18 46 (13/28): 24
Rajeswari ^24^, 1995, India	RCT	108 (C + A)	9: 100 ^4^	100	90 (97/108)	22 (20/91): 9 12 (11/89): 24
Ravenscroft ^71^, 2001, South Africa	Prospective observational	34 (C)	≥6: 100 12: 6	N/A	N/A	44 (14/32): 6 ^7^
Shah, 2016 ^78^, India	Prospective observational	28 (C + A)	≥12: 100 18-24: 17 ^8^	79	N/A	17 (4/24): 12 13 (3/24): 24
Shah, 2019 ^11^, India	Case series	6 (C)	23-32: 100	83	83 (5/6)	83 (5/6): >24
Tandon ^85^, 1985, India	Retrospective observational	50 (C + A)	12-18: 98	N/A	78 (39/50)	40 (20/50): N/A
Wasay ^56^, 2004, Pakistan	Retrospective observational	102 (C + A)	9-12: 100 ^4^	79 ^4^	34 (17/50)	NA
Yaramis ^86^, 1998, Turkey	Retrospective observational	4 (C)	12: 100 24: 50	100	100 (4/4)	N/A

Abbreviations: n, number; ATT, antituberculous therapy; N, number with known data; F/U, follow-up, C, children; A, adults; N/A, data not available; RCT, randomized controlled trial

^1^ All studies included HIV-uninfected patients or patients with unknown HIV status, except studies by Man **et al.** ^46^ and Marais **et al.** ^12^, that included 7, and 47 HIV-infected patients, respectively;

^2^ The definition varies between studies and include descriptions such as “complete recovery”, “no neurological disability”, “asymptomatic” and unspecified “good clinical recovery”. Several studies included patients with co-existing tuberculous meningitis that might have influenced clinical outcomes.

^3^ Including 30 patients with available data

^4^ Including patients followed up for at least 9 months

^5^ Including patients treated medically without surgical intervention

^6^ Excluding 1 patient who died during therapy

^7^ “32” refers to number of meningeal tuberculomas in 25 patients

^8^ Including patients followed up for at least 12 months

### Rationale for using longer versus shorter regimens

It is currently unknown whether persistent radiological enhancement of intracranial tuberculomas after 9–12 months of appropriate treatment represents active disease, inflammatory response in a sterilized lesion or merely revascularization. Additionally, a lack of radiological response could indicate that the diagnosis of tuberculoma is incorrect, particularly if the patient did not respond clinically to TB treatment, and alternative diagnoses should be considered. The consequences of stopping TB treatment prior to complete radiological resolution of intracranial tuberculous mass has rarely been explored. These important issues were discussed at the 3 ^rd^ International TBM Consortium meeting. Most clinicians were of the opinion that the continued enhancement does not necessarily represent treatment failure and that prolonged TB therapy (beyond 9–12 months) is not warranted in patients suspected of infection with or with proven *M.tb* strains susceptible to first-line drugs. This position is supported by the asymptomatic state of many patients and the paucity of AFB on staining and sterility of tuberculoma biopsy samples obtained prior to and following TB treatment initiation ^23, 82^. Immunohistochemical staining of excised tuberculomas also demonstrates high expression of vascular endothelial growth factor (VEGF) in the lesions with intense positivity of inflammatory mononuclear cells as well as reactive astrocytes and fibrocytes ^90^. The VEGF-induced angiogenesis in the granuloma capsule may therefore contribute, in addition to inflammation, to the persistent and prolonged contrast enhancement frequently seen on serial brain imaging. Furthermore, one trial reports no clinical or radiological deterioration at 24 months follow-up in 20 patients with persistent intracranial tuberculomas after completion of 9 months’ TB therapy ^24^.

A theoretical argument in favor of continuing treatment longer than 9–12 months is that drug penetration into the CNS is suboptimal and is likely even more suboptimal into the tuberculoma or tuberculous abscess. Drug penetration into cerebrospinal fluid is poor for rifampicin, the key sterilizing drug ^91^. Tuberculous abscesses that, unlike tuberculomas, are teeming with bacilli may potentially act as an immune sanctuary protecting the bacilli from immune effector cells within pus ^21^. The consequence of these factors may be that sterilization is not always achieved with 9–12 months treatment and that a longer duration may be required. The inability to obtain specimens to confirm sterilization make this an area of uncertainty. Pertinent, too, is that relapse of CNS TB could have catastrophic consequences. Furthermore, some patients need late re-initiation of immunomodulatory treatment and this should ideally be done while on TB treatment to avoid relapse resulting from iatrogenic immunosuppression. However, if treatment is continued because of residual lesions, when does the clinician stop therapy? Should this be until all contrast enhancing lesions have resolved – which can take years – or some arbitrary timepoint before then? A further question pertains to the timepoints at which repeat imaging should be performed, for which there are also no clear guidelines.

## Conclusion

Intracranial tuberculoma represents a major health concern in developing countries. Routine practices often include prescription of TB therapy until lesional enhancement has resolved, which may expose some patients to an unnecessarily prolonged treatment course. Because of the lack of evidence-based guidelines and equipoise with respect to shorter versus longer duration regimens, further research is needed. In the first instance, a multi-country audit of existing practice and outcomes in terms of cure and relapse would help in defining the spectrum of current practice. Ultimately, a randomized controlled trial comparing a standardized duration of TB treatment with duration based on brain imaging would provide a definitive answer to this question.

## Ethics statement

Images presented in Figure 1 and Figure 2 were obtained during a retrospective study of patients who presented with intracranial tuberculoma to Inkosi Albert Luthuli Central Hospital in Durban, South Africa. The Biomedical Research Ethics Committee (BREC) of the University of KwaZulu-Natal approved the study (BREC class approval number BCA325/15). Images presented in Figure 3 were obtained during a retrospective study of patients with tuberculous meningitis at Tygerberg Hospital in Cape Town, South Africa. The Health Research Ethics Committee of the University of Stellenbosch approved the study (N12/07/041). As these studies were retrospective folder reviews, and data were analyzed anonymously outside of the clinical settings, the ethics committees waived the requirement for informed consent and informed consent was not obtained.

## Data availability

### Underlying data

No data is associated with this article.

## References

[1] Dastur DK Manghani DK Udani PM: Pathology and pathogenetic mechanisms in neurotuberculosis. *Radiol Clin North Am.* 1995;33(4):733–52. 7610242

[2] Dastur HM: Diagnosis and neurosurgical treatment of tuberculous disease of the CNS. *Neurosurg Rev.* 1983;6(3):111–7. 10.1007/BF01742762 6371589

[3] Garg RK Desai P Kar M: Multiple ring enhancing brain lesions on computed tomography: an Indian perspective. *J Neurol Sci.* 2008;266(1–2):92–6. 10.1016/j.jns.2007.09.012 17945258

[4] Bhigjee AI Naidoo K Patel VB: Intracranial mass lesions in HIV-positive patients--the KwaZulu/Natal experience. Neuroscience AIDS Research Group. *S Afr Med J.* 1999;89(12):1284–8. 10678199

[5] Choe PG Park WB Song JS: Spectrum of intracranial parenchymal lesions in patients with human immunodeficiency virus infection in the Republic of Korea. *J Korean Med Sci.* 2010;25(7):1005–10. 10.3346/jkms.2010.25.7.1005 20592890 PMC2890875

[6] Modi M Mochan A Modi G: Management of HIV-associated focal brain lesions in developing countries. *QJM.* 2004;97(7):413–21. 10.1093/qjmed/hch080 15208429

[7] Smego RA Jr Orlovic D Wadula J: An algorithmic approach to intracranial mass lesions in HIV/AIDS. *Int J STD AIDS.* 2006;17(4):271–6. 10.1258/095646206776253390 16595052

[8] Gupta M Bajaj BK Khwaja G: Paradoxical response in patients with CNS tuberculosis. *J Assoc Physicians India.* 2003;51:257–60. 12839346

[9] Nair BR Rajshekhar V: Factors Predicting the Need for Prolonged (>24 Months) Antituberculous Treatment in Patients with Brain Tuberculomas. *World Neurosurg.* 2019;125:e236–e247. 10.1016/j.wneu.2019.01.053 30684718

[10] Rajshekhar V: Surgery for brain tuberculosis: a review. *Acta Neurochir (Wien).* 2015;157(10):1665–78. 10.1007/s00701-015-2501-x 26170188

[11] Shah I Shetty NS: Duration of anti-tuberculous therapy in children with persistent tuberculomas. *SAGE Open Med Case Rep.* 2019;7: 2050313X18823092. 10.1177/2050313X18823092 30671250 PMC6329029

[12] Marais S Roos I Mitha A: Presentation and outcome of patients with intracranial tuberculoma in a high HIV prevalence setting. *Int J Tuberc Lung Dis.* 2020; [ *In press*]. 10.5588/ijtld.19.0386 32127108

[13] Rich AR McCordock HA: The pathogenesis of tuberculous meningitis. *Bull Johns Hopkins Hosp.* 1933;52:2–37.

[14] Donald PR Schaaf HS Schoeman JF: Tuberculous meningitis and miliary tuberculosis: the Rich focus revisited. *J Infect.* 2005;50(3):193–5. 10.1016/j.jinf.2004.02.010 15780412

[15] Jain SK Paul-Satyaseela M Lamichhane G: *Mycobacterium tuberculosis* invasion and traversal across an *in vitro* human blood-brain barrier as a pathogenic mechanism for central nervous system tuberculosis. *J Infect Dis.* 2006;193(9):1287–95. 10.1086/502631 16586367

[16] Krishnan N Robertson BD Thwaites G: The mechanisms and consequences of the extra-pulmonary dissemination of *Mycobacterium tuberculosis*. *Tuberculosis (Edinb).* 2010;90(6):361–6. 10.1016/j.tube.2010.08.005 20829117

[17] Davis AG Rohlwink UK Proust A: The pathogenesis of tuberculous meningitis. *J Leukoc Biol.* 2019;105(2):267–80. 10.1002/JLB.MR0318-102R 30645042 PMC6355360

[18] Tsenova L Bergtold A Freedman VH: Tumor necrosis factor alpha is a determinant of pathogenesis and disease progression in mycobacterial infection in the central nervous system. *Proc Natl Acad Sci U S A.* 1999;96(10):5657–62. 10.1073/pnas.96.10.5657 10318940 PMC21916

[19] Dastur DK Dave UP: Ultrastructural basis of the vasculopathy in and around brain tuberculomas. Possible significance of altered basement membrane. *Am J Pathol.* 1977;89(1):35–50. 333937 PMC2032197

[20] Kumar R Pandey CK Bose N: Tuberculous brain abscess: clinical presentation, pathophysiology and treatment (in children). *Childs Nerv Syst.* 2002;18(3–4):118–23. 10.1007/s00381-002-0575-2 11981617

[21] Bernaerts A Vanhoenacker FM Parizel PM: Tuberculosis of the central nervous system: overview of neuroradiological findings. *Eur Radiol.* 2003;13(8):1876–90. 10.1007/s00330-002-1608-7 12942288

[22] Arseni C: Two hundred and one cases of intracranial tuberculoma treated surgically. *J Neurol Neurosurg Psychiatry.* 1958;21(4):308–11. 10.1136/jnnp.21.4.308 13611575 PMC497336

[23] Poonnoose SI Rajshekhar V: Rate of resolution of histologically verified intracranial tuberculomas. *Neurosurgery.* 2003;53(4):873–8. 10.1227/01.neu.0000083553.25421.6f 14519219

[24] Rajeswari R Sivasubramanian S Balambal R: A controlled clinical trial of short-course chemotherapy for tuberculoma of the brain. *Tuber Lung Dis.* 1995;76(4):311–7. 10.1016/S0962-8479(05)80029-2 7579312

[25] Deogaonkar M De R Sil K: Pituitary tuberculosis presenting as pituitary apoplexy. *Int J Infect Dis.* 2006;10(4):338–9. 10.1016/j.ijid.2005.05.008 16413219

[26] Kalita J Ranjan P Misra UK: Hemichorea: a rare presentation of tuberculoma. *J Neurol Sci.* 2003;208(1–2):109–11. 10.1016/s0022-510x(02)00417-3 12639734

[27] Azeemuddin M Alvi A Sayani R: Neuroimaging Findings in Tuberculosis: A Single-Center Experience in 559 Cases. *J Neuroimaging.* 2019;29(5):657–668. 10.1111/jon.12627 31115112

[28] Sonmez G Ozturk E Sildiroglu HO: MRI findings of intracranial tuberculomas. *Clin Imaging.* 2008;32(2):88–92. 10.1016/j.clinimag.2007.08.024 18313571

[29] Patkar D Narang J Yanamandala R: Central nervous system tuberculosis: pathophysiology and imaging findings. *Neuroimaging Clin N Am.* 2012;22(4):677–705. 10.1016/j.nic.2012.05.006 23122262

[30] van Toorn R du Plessis AM Schaaf HS: Clinicoradiologic response of neurologic tuberculous mass lesions in children treated with thalidomide. *Pediatr Infect Dis J.* 2015;34(2):214–8. 10.1097/INF.0000000000000539 25741973

[31] Omuro AM Leite CC Mokhtari K: Pitfalls in the diagnosis of brain tumours. *Lancet Neurol.* 2006;5(11):937–948. 10.1016/S1474-4422(06)70597-X 17052661

[32] Garg RK Sinha MK: Multiple ring-enhancing lesions of the brain. *J Postgrad Med.* 2010;56(4):307–316. 10.4103/0022-3859.70939 20935408

[33] Gupta RK Husain N Kathuria MK: Magnetization transfer MR imaging correlation with histopathology in intracranial tuberculomas. *Clin Radiol.* 2001;56(8):656–663. 10.1053/crad.2001.0752 11467867

[34] Chaudhary V Bano S Garga UC: Central Nervous System Tuberculosis: An Imaging Perspective. *Can Assoc Radiol J.* 2017;68(2):161–170. 10.1016/j.carj.2016.10.007 28283299

[35] Kong A Koukourou A Boyd M: Metastatic adenocarcinoma mimicking 'target sign' of cerebral tuberculosis. *J Clin Neurosci.* 2006;13(9):955–958. 10.1016/j.jocn.2005.11.039 17085301

[36] Gasparetto EL Cabral RF da Cruz LC Jr: Diffusion imaging in brain infections. *Neuroimaging Clin N Am.* 2011;21(1):89–113, viii. 10.1016/j.nic.2011.01.011 21477753

[37] Batra A Tripathi RP: Diffusion-weighted magnetic resonance imaging and magnetic resonance spectroscopy in the evaluation of focal cerebral tubercular lesions. *Acta Radiol.* 2004;45(6):679–688. 10.1080/02841850410001169 15587429

[38] Gupta RK Prakash M Mishra AM: Role of diffusion weighted imaging in differentiation of intracranial tuberculoma and tuberculous abscess from cysticercus granulomas-a report of more than 100 lesions. *Eur J Radiol.* 2005;55(3):384–392. 10.1016/j.ejrad.2005.02.003 16129246

[39] Parry AH Wani AH Shaheen FA: Evaluation of intracranial tuberculomas using diffusion-weighted imaging (DWI), magnetic resonance spectroscopy (MRS) and susceptibility weighted imaging (SWI). *Br J Radiol.* 2018;91(1091):20180342. 10.1259/bjr.20180342 29987985 PMC6475934

[40] Lai PH Hsu SS Ding SW: Proton magnetic resonance spectroscopy and diffusion-weighted imaging in intracranial cystic mass lesions. *Surg Neurol.* 2007;68 Suppl 1:S25–36. 17963918 10.1016/j.surneu.2007.07.080

[41] Alam MS Sajjad Z Azeemuddin M: Diffusion weighted MR imaging of ring enhancing brain lesions. *J Coll Physicians Surg Pak.* 2012;22(7):428–431. 22747861

[42] Anuradha HK Garg RK Sinha MK: Intracranial tuberculomas in patients with tuberculous meningitis: predictors and prognostic significance. *Int J Tuberc Lung Dis.* 2011;15(2):234–9. 21219687

[43] Awada A Daif AK Pirani M: Evolution of brain tuberculomas under standard antituberculous treatment. *J Neurol Sci.* 1998;156(1):47–52. 10.1016/s0022-510x(98)00024-0 9559986

[44] Haris M Gupta RK Husain M: Assessment of therapeutic response in brain tuberculomas using serial dynamic contrast-enhanced MRI. *Clin Radiol.* 2008;63(5):562–74. 10.1016/j.crad.2007.11.002 18374721

[45] Kalita J Prasad S Misra UK: Predictors of paradoxical tuberculoma in tuberculous meningitis. *Int J Tuberc Lung Dis.* 2014;18(4):486–91. 10.5588/ijtld.13.0556 24670707

[46] Man H Sellier P Boukobza M: Central nervous system tuberculomas in 23 patients. *Scand J Infect Dis.* 2010;42(6–7):450–4. 10.3109/00365541003598999 20297925

[47] Ranjan P Kalita J Misra UK: Serial study of clinical and CT changes in tuberculous meningitis. *Neuroradiology.* 2003;45(5):277–82. 10.1007/s00234-003-0958-4 12687301

[48] Tai ML Nor HM Kadir KA: Paradoxical Manifestation is Common in HIV-negative Tuberculous Meningitis. *Medicine (Baltimore).* 2016;95(1):e1997. 10.1097/MD.0000000000001997 26735523 PMC4706243

[49] Unal A Sutlas PN: Clinical and radiological features of symptomatic central nervous system tuberculomas. *Eur J Neurol.* 2005;12(10):797–804. 10.1111/j.1468-1331.2005.01067.x 16190918

[50] Vidal JE Hernández AV Oliveira AC: Cerebral tuberculomas in AIDS patients: a forgotten diagnosis? *Arq Neuropsiquiatr.* 2004;62(3B):793–6. 10.1590/s0004-282x2004000500010 15476071

[51] Lesprit P Zagdanski AM de La Blanchardiere A: Cerebral tuberculosis in patients with the acquired immunodeficiency syndrome (AIDS). Report of 6 cases and review. *Medicine (Baltimore).* 1997;76(6):423–31. 10.1097/00005792-199711000-00005 9413428

[52] Nicolls DJ King M Holland D: Intracranial tuberculomas developing while on therapy for pulmonary tuberculosis. *Lancet Infect Dis.* 2005;5(12):795–801. 10.1016/S1473-3099(05)70299-1 16310151

[53] Schoeman JF Fieggen G Seller N: Intractable intracranial tuberculous infection responsive to thalidomide: report of four cases. *J Child Neurol.* 2006;21(4):301–8. 10.1177/08830738060210040801 16900926

[54] van Toorn R Rabie H Dramowski A: Neurological manifestations of TB-IRIS: a report of 4 children. *Eur J Paediatr Neurol.* 2012;16(6):676–82. 10.1016/j.ejpn.2012.04.005 22658306

[55] Jain SK Kwon P Moss WJ: Management and outcomes of intracranial tuberculomas developing during antituberculous therapy: case report and review. *Clin Pediatr (Phila).* 2005;44(5):443–50. 10.1177/000992280504400510 15965552

[56] Wasay M Moolani MK Zaheer J: Prognostic indicators in patients with intracranial tuberculoma: a review of 102 cases. *J Pak Med Assoc.* 2004;54(2):83–7. 15134209

[57] Pauranik A Behari M Maheshwari MC: Appearance of tuberculoma during treatment of tuberculous meningitis. *Jpn J Med.* 1987;26(3):332–4. 10.2169/internalmedicine1962.26.332 3320427

[58] Singh AK Malhotra HS Garg RK: Paradoxical reaction in tuberculous meningitis: presentation, predictors and impact on prognosis. *BMC Infect Dis.* 2016;16:306. 10.1186/s12879-016-1625-9 27329253 PMC4915108

[59] Narita M Ashkin D Hollender ES: Paradoxical worsening of tuberculosis following antiretroviral therapy in patients with AIDS. *Am J Respir Crit Care Med.* 1998;158(1):157–61. 10.1164/ajrccm.158.1.9712001 9655723

[60] Brown CS Smith CJ Breen RA: Determinants of treatment-related paradoxical reactions during anti-tuberculosis therapy: a case control study. *BMC Infect Dis.* 2016;16:479. 10.1186/s12879-016-1816-4 27600661 PMC5013570

[61] Marais S Meintjes G Pepper DJ: Frequency, severity, and prediction of tuberculous meningitis immune reconstitution inflammatory syndrome. *Clin Infect Dis.* 2013;56(3):450–60. 10.1093/cid/cis899 23097584 PMC3540040

[62] Walker NF Stek C Wasserman S: The tuberculosis-associated immune reconstitution inflammatory syndrome: recent advances in clinical and pathogenesis research. *Curr Opin HIV AIDS.* 2018;13(6):512–21. 10.1097/COH.0000000000000502 30124473 PMC6181275

[63] Schoeman JF Van Zyl LE Laubscher JA: Effect of corticosteroids on intracranial pressure, computed tomographic findings, and clinical outcome in young children with tuberculous meningitis. *Pediatrics.* 1997;99(2):226–31. 10.1542/peds.99.2.226 9024451

[64] Meintjes G Wilkinson RJ Morroni C: Randomized placebo-controlled trial of prednisone for paradoxical tuberculosis-associated immune reconstitution inflammatory syndrome. *AIDS.* 2010;24(15):2381–90. 10.1097/QAD.0b013e32833dfc68 20808204 PMC2940061

[65] Meintjes G Stek C Blumenthal L: Prednisone for the Prevention of Paradoxical Tuberculosis-Associated IRIS. *N Engl J Med.* 2018;379(20):1915–25. 10.1056/NEJMoa1800762 30428290

[66] Shah I Borse S: Paradoxical tuberculomas after completion of antituberculous treatment. *Trop Med Health.* 2012;40(1):15–17. 22949802 10.2149/tmh.2012-09 PMC3426828

[67] Machida A Ishihara T Amano E: Late-onset paradoxical reactions 10 years after treatment for tuberculous meningitis in an HIV-negative patient: a case report. *BMC Infect Dis.* 2018;18(1):313. 10.1186/s12879-018-3229-z 29980175 PMC6035426

[68] World Health Organization: Treatment of tuberculosis guidelines. Fourth edition.2010; Accessed: Sept 09 2019. Reference Source 23741786

[69] Nahid P Dorman SE Alipanah N: Official American Thoracic Society/Centers for Disease Control and Prevention/Infectious Diseases Society of America Clinical Practice Guidelines: Treatment of Drug-Susceptible Tuberculosis. *Clin Infect Dis.* 2016;63(7):e147–e95. 10.1093/cid/ciw376 27516382 PMC6590850

[70] Thwaites G Fisher M Hemingway C: British Infection Society guidelines for the diagnosis and treatment of tuberculosis of the central nervous system in adults and children. *J Infect.* 2009;59(3):167–87. 10.1016/j.jinf.2009.06.011 19643501

[71] Ravenscroft A Schoeman JF Donald PR: Tuberculous granulomas in childhood tuberculous meningitis: radiological features and course. *J Trop Pediatr.* 2001;47(1):5–12. 10.1093/tropej/47.1.5 11245351

[72] Donald PR Van Toorn R: Use of corticosteroids in tuberculous meningitis. *Lancet.* 2016;387(10038):2585–7. 10.1016/S0140-6736(16)30770-X 27353808

[73] Blackmore TK Manning L Taylor WJ: Therapeutic use of infliximab in tuberculosis to control severe paradoxical reaction of the brain and lymph nodes. *Clin Infect Dis.* 2008;47(10):e83–85. 10.1086/592695 18840076

[74] Coulter JB Baretto RL Mallucci CL: Tuberculous meningitis: protracted course and clinical response to interferon-gamma. *Lancet Infect Dis.* 2007;7(3):225–232. 10.1016/S1473-3099(07)70054-3 17317604

[75] Akhaddar A: Surgical therapy.In *“Tuberculosis Of The Central Nervous System: Pathogenesis, Imaging, And Management”*eds M. Turgut, A. Akhaddar, A.T. Turgut, R.K. Garg. Springer.2017;173–191. 10.1007/978-3-319-50712-5_14

[76] Schoeman JF Van Zyl LE Laubscher JA: Serial CT scanning in childhood tuberculous meningitis: prognostic features in 198 cases. *J Child Neurol.* 1995;10(4):320–9. 10.1177/088307389501000417 7594269

[77] Thwaites GE Macmullen-Price J Tran TH: Serial MRI to determine the effect of dexamethasone on the cerebral pathology of tuberculous meningitis: an observational study. *Lancet Neurol.* 2007;6(3):230–6. 10.1016/S1474-4422(07)70034-0 17303529 PMC4333204

[78] Shah IA Asimi RP Kawoos Y: Tuberculomas of the brain with and without associated meningitis: a cohort of 28 cases treated with anti-tuberculosis drugs at a tertiary care centre. *International Journal of Contemporary Medical Research.* 2016;3(12):3484–7. Reference Source

[79] Idris MN Sokrab TE Arbab MA: Tuberculoma of the brain: a series of 16 cases treated with anti-tuberculosis drugs. *Int J Tuberc Lung Dis.* 2007;11(1):91–5. 17217136

[80] Li H Liu W You C: Central nervous system tuberculoma. *J Clin Neurosci.* 2012;19(5):691–5. 10.1016/j.jocn.2011.05.045 22398188

[81] Afghani B Lieberman JM: Paradoxical enlargement or development of intracranial tuberculomas during therapy: case report and review. *Clin Infect Dis.* 1994;19(6):1092–9. 10.1093/clinids/19.6.1092 7888539

[82] Bayindir C Mete O Bilgic B: Retrospective study of 23 pathologically proven cases of central nervous system tuberculomas. *Clin Neurol Neurosurg.* 2006;108(4):353–7. 10.1016/j.clineuro.2005.03.001 16644403

[83] Gupta RK Jena A Singh AK: Role of magnetic resonance (MR) in the diagnosis and management of intracranial tuberculomas. *Clin Radiol.* 1990;41(2):120–7. 10.1016/s0009-9260(05)80143-6 2306912

[84] Harder E Al-Kawi MZ Carney P: Intracranial tuberculoma: conservative management. *Am J Med.* 1983;74(4):570–6. 10.1016/0002-9343(83)91011-2 6837585

[85] Tandon PN Bhargava S: Effect of medical treatment on intracranial tuberculoma--a CT study. *Tubercle.* 1985;66(2):85–97. 10.1016/0041-3879(85)90073-x 4024278

[86] Yaramis A Gurkan F Elevli M: Central nervous system tuberculosis in children: a review of 214 cases. *Pediatrics.* 1998;102(5):E49. 10.1542/peds.102.5.e49 9794979

[87] Monteiro R Carneiro JC Costa C: Cerebral tuberculomas - A clinical challenge. *Respir Med Case Rep.* 2013;9:34–7. 10.1016/j.rmcr.2013.04.003 26029627 PMC3949551

[88] Jinkins JR: Computed tomography of intracranial tuberculosis. *Neuroradiology.* 1991;33(2):126–35. 10.1007/bf00588250 2046896

[89] Wasay M: Central nervous system tuberculosis and paradoxical response. *South Med J.* 2006;99(4):331–2. 10.1097/01.smj.0000209231.88651.07 16634237

[90] Husain N Awasthi S Haris M: Vascular endothelial growth factor as a marker of disease activity in neurotuberculosis. *J Infect.* 2008;56(2):114–9. 10.1016/j.jinf.2007.11.004 18158186

[91] Wasserman S Davis A Wilkinson RJ: Key considerations in the pharmacotherapy of tuberculous meningitis. *Expert Opin Pharmacother.* 2019;20(15):1–5. 10.1080/14656566.2019.1638912 31305179 PMC6763362

