# Supplementary material for: Management of intracranial tuberculous mass lesions: how long should we treat for?
Source: Wellcome Open Res. 2020 Feb 26;4:158. Originally published 2019 Oct 15. [Version 3] doi: 10.12688/wellcomeopenres.15501.3 (PMC6996525; doi:10.12688/wellcomeopenres.15501.3)
Supplement: Peer review history

# Peer review: Reviewer response for version 3

**DOI:** 10.21956/wellcomeopenres.17274.r38036

**Published:** 2020-03-04

**Authors:** Julie Higashi

**Affiliations:** 1 Tuberculosis Control Program, Division of Dental and Medical Affairs, Los Angeles County Department of Public Health, Los Angeles, CA, USA; 2 Francis J Curry International Tuberculosis Center, University of California, San Francisco, San Francisco, CA, USA

I have reviewed the revised manuscript and appreciate the authors careful attention to my suggestions. I have no further comments. Thank you for bringing attention to the challenges of managing CNS TB.

I confirm that I have read this submission and believe that I have an appropriate level of expertise to confirm that it is of an acceptable scientific standard.

# Peer review: Reviewer response for version 3

**DOI:** 10.21956/wellcomeopenres.17274.r38037

**Published:** 2020-02-28

**Authors:** Jerome H. Chin

**Affiliations:** 1 Department of Neurology, NYU Langone Health, New York City, NY, USA

The revised article provides an improved discussion of the neuroimaging features of tuberculomas and tubercular abscesses and the differential diagnosis of enhancing intracranial mass lesions.

I confirm that I have read this submission and believe that I have an appropriate level of expertise to confirm that it is of an acceptable scientific standard.

# Peer review: Reviewer response for version 2

**DOI:** 10.21956/wellcomeopenres.17008.r37110

**Published:** 2020-01-31

**Authors:** Julie Higashi

**Affiliations:** 1 Tuberculosis Control Program, Division of Dental and Medical Affairs, Los Angeles County Department of Public Health, Los Angeles, CA, USA; 2 Francis J Curry International Tuberculosis Center, University of California, San Francisco, San Francisco, CA, USA

The article presents the current clinical approach to the management of tuberculous intracranial mass lesions, which is variable because of limited data that inform treatment interventions that might optimize outcomes.

 Overall, the article generally presents a helpful review of the status of this problem and I agree with the recommendations for management, but could be improved by the following:

 While corticosteroids remain the mainstay of immunomodulatory therapy, other agents besides thalidomide may improve outcomes. There are few reports of other agents improving outcomes (e.g. infliximab - Blackmore (2008) ^1^, interferon gamma - Coulter (2007) ^2^), and suggestion that other agents (anakinra) may be beneficial, but the future of immunomodulatory therapy with more targeted agents is an exciting new area. A line or two with references to this handful of case reports as another avenue of investigation would make the article more complete.

 Please include recommendations regarding monitoring of patients post treatment (imaging frequency and duration of follow up) - and frequency of recurrent paradoxical response events or symptoms after treatment that are not thought to be treatment failure. Other clinicians with much experience treating CNS TB who have anecdotally describe patients having focal neurological deficits that don't represent relapse of TB, but immune response years after treatment completion. If this is something that the panel of experts have noted, even if infrequent, is worth mentioning.

I confirm that I have read this submission and believe that I have an appropriate level of expertise to confirm that it is of an acceptable scientific standard, however I have significant reservations, as outlined above.

## References

[1] : Therapeutic use of infliximab in tuberculosis to control severe paradoxical reaction of the brain and lymph nodes. *Clin Infect Dis*.2008;47(10) : 10.1086/592695 e83-5 10.1086/592695 18840076

[2] : Tuberculous meningitis: protracted course and clinical response to interferon-gamma. *The Lancet Infectious Diseases*.2007;7(3) : 10.1016/S1473-3099(07)70054-3 225-232 10.1016/S1473-3099(07)70054-3 17317604

# Response

**Published:** 2020-02-21

**Authors:** Suzaan Marais

**Affiliations:** University of Cape Town, South Africa

While corticosteroids remain the mainstay of immunomodulatory therapy, other agents besides thalidomide may improve outcomes. There are few reports of other agents improving outcomes (e.g. infliximab - Blackmore (2008) ^1^, interferon gamma - Coulter (2007) ^2^), and suggestion that other agents (anakinra) may be beneficial, but the future of immunomodulatory therapy with more targeted agents is an exciting new area. A line or two with references to this handful of case reports as another avenue of investigation would make the article more complete.

1. Blackmore TK, Manning L, Taylor WJ, Wallis RS: Therapeutic use of infliximab in tuberculosis to control severe paradoxical reaction of the brain and lymph nodes. *Clin Infect Dis*. 2008; **47**(10): e83-5 PubMed Abstract | Publisher Full Text 

2. Coulter J, Baretto R, Mallucci C, Romano M, Abernethy L, Isherwood D, Kumararatne D, Lammas D: Tuberculous meningitis: protracted course and clinical response to interferon-gamma. *The Lancet Infectious Diseases*. 2007; **7**(3): 225-232 Publisher Full Text 

**RESPONSE:** We have added a brief description of some additional immunomodulatory agents that have shown anecdotal benefit in paradoxical TB reactions affecting the CNS and emphasized the need for future studies investigating the utility of host-directed therapies in these patients. 

Please include recommendations regarding monitoring of patients post treatment (imaging frequency and duration of follow up) - and frequency of recurrent paradoxical response events or symptoms after treatment that are not thought to be treatment failure. Other clinicians with much experience treating CNS TB who have anecdotally describe patients having focal neurological deficits that don't represent relapse of TB, but immune response years after treatment completion. If this is something that the panel of experts have noted, even if infrequent, is worth mentioning.

**RESPONSE:** We have added a few sentences on the variability of follow-up practices in patients with intracranial tuberculous mass lesions as well as the lack of clear guidelines for timing of follow-up imaging in patients with persistent lesions. We reference case reports of patients with recurrent lesions after completion of TB treatment and comment on the various potential reasons for such recurrences, including paradoxical reaction.

# Peer review: Reviewer response for version 2

**DOI:** 10.21956/wellcomeopenres.17008.r37112

**Published:** 2019-12-18

**Authors:** Benedict Michael

**Affiliations:** 1 Institute of Infection and Global Health, University of Liverpool, Liverpool, L69 7BE, UK

In this excellent and thorough overview, the authors accurately summarise the clinical presentation, neuroimaging findings, and pathogenesis of CNS TB, and focus on the data (and lack thereof) to guide the duration of TB therapy.

 The manuscript would be strengthened if, in addition to the serial MRI scans in one patient, a figure was included to demonstrate the specific neuroimaging findings described in the text (Figure allowance permitting). This is of particular interest given that they describe, at least theoretical, rationale for cessation/continuing TB therapy based on the nature of the lesions.

 The manuscript (space permitting) would also benefit from a more descriptive outline of the nature of the RCT investigating TB therapy duration proposed by these authors, who represent leaders in the field. For example, inclusion/exclusion, how to address HIV/ART status, imaging and clinical sub-group analyses, and controlling for corticosteroid use. This would be in the discussion as opposed to the conclusion.

I confirm that I have read this submission and believe that I have an appropriate level of expertise to confirm that it is of an acceptable scientific standard.

# Response

**Published:** 2020-02-21

**Authors:** Suzaan Marais

**Affiliations:** University of Cape Town, South Africa

In this excellent and thorough overview, the authors accurately summarise the clinical presentation, neuroimaging findings, and pathogenesis of CNS TB, and focus on the data (and lack thereof) to guide the duration of TB therapy.

**RESPONSE**: We thank the reviewer for this comment.

The manuscript would be strengthened if, in addition to the serial MRI scans in one patient, a figure was included to demonstrate the specific neuroimaging findings described in the text (Figure allowance permitting). This is of particular interest given that they describe, at least theoretical, rationale for cessation/continuing TB therapy based on the nature of the lesions.

**RESPONSE:** We have added additional images showing various stages of intracranial tuberculous mass lesions (Figures 1 and 3). 

The manuscript (space permitting) would also benefit from a more descriptive outline of the nature of the RCT investigating TB therapy duration proposed by these authors, who represent leaders in the field. For example, inclusion/exclusion, how to address HIV/ART status, imaging and clinical sub-group analyses, and controlling for corticosteroid use. This would be in the discussion as opposed to the conclusion.

**RESPONSE:** A RCT investigating TB therapy duration will, as the reviewer rightly points out, require careful consideration of multiple potential confounders. Such a detailed description is outside the scope of this manuscript but could be the subject of a future manuscript.

# Peer review: Reviewer response for version 2

**DOI:** 10.21956/wellcomeopenres.17008.r36913

**Published:** 2019-11-19

**Authors:** Jerome H. Chin

**Affiliations:** 1 Department of Neurology, NYU Langone Health, New York City, NY, USA

This manuscript reviews published studies of the diagnosis and treatment of intracranial tuberculomas and provides a consensus opinion on current practices and research needs.

 The authors state “The diagnosis of intracranial tuberculoma is most often presumptive and based on radiological features, supportive evidence of TB elsewhere and response to TB treatment.” Since the diagnosis of intracranial tuberculoma rests in part on exclusion of other causes of intracranial mass lesions, it is necessary for the authors to discuss neuroimaging features (including DWI/ADC) that may or may not distinguish tuberculomas and tubercular abscesses from other causes of rim-enhancing and homogenous-enhancing lesions, including metastases, sarcoidosis, lymphoma, and bacterial, fungal and parasitic infections (e.g. staphylococcus, brucella, cryptococcus, aspergillus, toxoplasma gondii, taenia solium, schistosoma). Further, the authors should discuss the possibility that lack of radiological improvement after > 12 months of anti-tuberculosis treatment could indicate that the diagnosis of tuberculoma is incorrect and should prompt consideration of alternative diagnoses.

I confirm that I have read this submission and believe that I have an appropriate level of expertise to confirm that it is of an acceptable scientific standard, however I have significant reservations, as outlined above.

# Response

**Published:** 2020-02-21

**Authors:** Suzaan Marais

**Affiliations:** University of Cape Town, South Africa

The authors state “The diagnosis of intracranial tuberculoma is most often presumptive and based on radiological features, supportive evidence of TB elsewhere and response to TB treatment.” Since the diagnosis of intracranial tuberculoma rests in part on exclusion of other causes of intracranial mass lesions, it is necessary for the authors to discuss neuroimaging features (including DWI/ADC) that may or may not distinguish tuberculomas and tubercular abscesses from other causes of rim-enhancing and homogenous-enhancing lesions, including metastases, sarcoidosis, lymphoma, and bacterial, fungal and parasitic infections (e.g. staphylococcus, brucella, cryptococcus, aspergillus, toxoplasma gondii, taenia solium, schistosoma). 

**RESPONSE:** We have added a paragraph that includes 1) a list of differential diagnoses for intracranial space-occupying lesions; 2) a finding on CT that is very suggestive of tuberculoma versus other etiologies; 3) a discussion of the utility of diffusion weighted imaging/apparent diffusion coefficient values in tuberculous mass lesions and 4) a note of other advanced imaging that are under investigation for tuberculous brain lesion diagnosis. 

Further, the authors should discuss the possibility that lack of radiological improvement after > 12 months of anti-tuberculosis treatment could indicate that the diagnosis of tuberculoma is incorrect and should prompt consideration of alternative diagnoses.

**RESPONSE:** We have included a sentence cautioning that a lack of radiological response to TB treatment could indicate that the diagnosis of tuberculoma was incorrect.
